# Gut Microbiota Host–Gene Interaction

**DOI:** 10.3390/ijms232213717

**Published:** 2022-11-08

**Authors:** Paola Cuomo, Rosanna Capparelli, Marco Alifano, Antonio Iannelli, Domenico Iannelli

**Affiliations:** 1 Department of Agriculture Sciences, University of Naples “Federico II”, Via Università 100, Portici, 80055 Naples, Italy; 2 Thoracic Surgery Department, Cochin Hospital, APHP Centre, University of Paris, 75014 Paris, France; 3 INSERM, U1138, Team “Cancer, Immune Control, and Escape”, Cordeliers Research Center, University of Paris, 75014 Paris, France; 4 Centre Hospitalier Universitaire de Nice—Digestive Surgery and Liver Transplantation Unit, Archet 2 Hospital, 06202 Nice, France; 5 Faculty of Medicine, Université Côte d’Azur, 06000 Nice, France; 6 INSERM, U1065, Team 8 “Hepatic Complications of Obesity and Alcohol”, 06204 Nice, France

**Keywords:** gut microbiota, gut cancer, lung cancer, *Helicobacter pylori*, inflammatory bowel disease

## Abstract

Studies carried out in the last ten years have shown that the metabolites made up from the gut microbiota are essential for multiple functions, such as the correct development of the immune system of newborns, interception of pathogens, and nutritional enrichment of the diet. Therefore, it is not surprising that alteration of the gut microbiota is the starting point of gastrointestinal infection, obesity, type 2 diabetes, inflammatory bowel disease, colorectal cancer, and lung cancer. Diet changes and antibiotics are the major factors damaging the gut microbiota. Early exposure of the newborns to antibiotics may prevent their correct development of the immune system, exposing them to pathogen infections, allergies, and chronic inflammatory diseases. We already know much on how host genes, microbiota, and the environment interact, owing to experiments in several model animals, especially in mice; advances in molecular technology; microbiota transplantation; and comparative metagenomic analysis. However, much more remains to be known. Longitudinal studies on patients undergoing to therapy, along with the identification of bacteria prevalent in responding patients may provide valuable data for improving therapies.

## 1. Introduction

**Figure 1:**
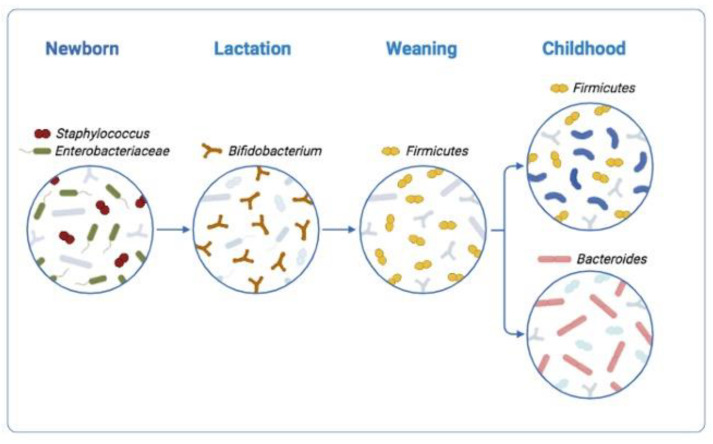
Gut microbiota development, from birth to childhood.

The long coevolution between the human host and the gut microbial community (gut microbiota) has established a strict interaction between them; the microbiota outnumbers somatic and germ cells of the human body 10-fold [1] and the number of the human genes 100-fold [2]. The gut microbiota is rich in genes that provide energy or encode enzymes involved in the carbohydrate metabolism, such as glycoside hydrolases and lyases [3]. Humans instead have few of these genes. Yet, humans consume diets rich in carbohydrates, including fibers. The solution to this puzzle comes from the cooperation between the two partners: the host providing glycans and the gut microbiota monosaccharides and disaccharides derived from the fiber present in the diet and not digested by the host. Finally, monosaccharides and disaccharides enrich of nutrients the diet of both: gut microbiota and host. Furthermore, the gut microbiota provides vitamin B12, biotin, and folic acid. B12 is particularly important since it mediates both host–microbe and microbe–microbe interactions [4]. The majority of the bacterial species encode multiple corrinoid transporters and corrinoid-dependent enzymes, but do not produce de novo corrinoids (molecules containing four pyrrole rings similar to porphyrins) [5]. Rather, corrinoids acquire and convert those produced by other bacteria present in the environment. Why do bacteria not synthetize de novo corrinoids? The answer is that bacteria expressing a corrinoid with a new ligand may gain a competitive edge in that niche over bacteria that do not express this ligand [6]. Experiments in gnotobiotic mice (mice colonized with specific microbial species) have confirmed in vivo (in a mammalian gut) the microbial fitness of single corrinoids. In conclusion, we expect that the differences between corrinoids—properly deciphered—might help to detect new biomarkers of environmental enteric dysfunctions [7] and help to better understand how corrinoids regulate gene expression in bacteria microbiota [8]. During the first year of life, in the gut microbiota of newborns several *Bifidobacterium* species prevail, able to metabolize the oligosaccharides present in the maternal milk [9]. Alternatively, after weaning *Firmicutes* and *Bacteroides* phyla prevail, which supervise the development of the adaptive immune system and provide nutrients to the microbiota [10] (Figure 1).

The early use of antibiotics can seriously damage the gut microbiota. Rather, following stress—diet change, a short time antibiotic treatment of an adult host, or a bacterial infection—generally the microbiota returns to a state of equilibrium. If these short-term changes interrupt the production of metabolites, the alteration of the involved host genes may be long lasting.

The gut microbiota produces molecules that protect the host against pathogens, favoring the interaction between host and gut microbiota. Furthermore, carbohydrates and proteins only partially degraded by the host support the growth of the microbiota. For instance, the microbiota degrades the gluten into amino acids, tryptophan in indole 3-acetate and tryptamine, and phospholipids in trimethylamine and choline. Since choline is a semi-essential component of the diet it cannot be completely excluded [11], it is rather recommended to reduce the intake of food rich in choline.

## 2. How Gut Microbiota Host–Gene Interactions Are Studied

A powerful way to investigate how microbiota and gene expression interact is to use model organisms: for example, comparing the results obtained with germ-free (GF) and conventionally raised (CR) mice. The same approach offers the opportunity to control the environment (including change of diet: the factor that most influences the microbiota composition), and to extend the study to multiple tissues. Mice and humans share 99% of their genes and several bacterial phyla of the gut microbiota [12]. These common properties make mice an excellent tool for detecting gene–gut microbiota interactions capable of being extended to humans. Additional animal models used for the same purpose are rats, zebrafish, and pigs, reared under germ-free conditions and transplanted with specific human gut microbial species. 

Transplantation of specific components of gut microbiota from diseased mice into germ-free (GF) healthy mice have demonstrated that several diseases—obesity, colitis, metabolic adiposity (accumulation of fat-favoring metabolic diseases)—can be transmitted via the gut microbiota. Concurrently, other studies have demonstrated that specific components of gut microbiota can resolve inflammatory bowel disease (IBD), while total transplantation of the microbiota is effective against colitis and Crohn’s disease [13]. Altogether, the above results show that the gut microbiota can have a positive or negative influence on the host’s health, depending upon the context. Use of GF mice also gives the opportunity to know whether the effect under study is attributable to the gut microbiota or not. If the effect of a treatment is absent in GF mice—but present in the CR mice—then the effect is attributable to the gut microbiota. While highly informative, the use of GF mice has a limitation: in GF mice, the absence of microbiota is associated with a very thin layer of mucous in the gastrointestinal tract compared to CR mice [14]. Thus, the researcher must exclude that this and other possible factors influence the results. In any case, GF mice demonstrate that the gut microbiota exerts a strong influence on host gene expression. Specifically, CR mice carrying the deletion of the intestinal epithelial cell-specific histone deacetylase 3 (*HDACDEL*) gene show altered composition of gut microbiota and intestinal damage, indicating that the *HDAC3* gene regulates intestinal homeostasis. The same HDACDEL mice—grown in GF conditions—show very attenuated intestinal damage. These results show that *HDAC3*—to maintain the intestinal homeostasis—must necessarily interact with the gut microbiota [15].

Direct evidence that antibiotics perturb the gut microbiota comes from vancomycin. In mice, this antibiotic causes enterococcus bacteremia, frequently observed in hospitalized patients. Many newborn infants are exposed to antibiotics, from parturition up to childhood through food supply. To know more about the effect of early exposure of children to antibiotics, young mice were given antibiotics—individual or in combination—and in the same dosage used for livestock. The study demonstrated that early exposure of children to antibiotics contributes to obesity, favors alterations in skeletal development, and insulin resistance [16]. To learn more about the clinical utility of this research area, the reader may consult the exhaustive work by AD Kostic et al. [17].

## 3. Bacteroides, the Microbial Species Predominant in the Human Gut Microbiota

The human gut microbiota secretes glycosaminoglycans (GAGs) and mucin, abundantly and independently from nutrients derived from the daily host diet. GAGs and mucin are assimilated from the gut microbiota, as demonstrated by the increased number of *Bacteroides* in the gut microbiota of mice, following chondroitin sulphate administration [18] and upregulation of the genes controlling the assimilation of GAGs, following GAGs administration [19]. *Bacteroides* grown in GAGs or mucin minimal medium secrete short chain fatty acids and most of the amino acids (including those absent in the host diet) in amounts close to the dose recommended by the World Health Organization (WHO). In addition to nutrients, *Bacteroides* species derive ℽ-amino butyrate (GABA) from GAGs. Deficit of GABA is associated with depressive symptoms [19]. Thus, *Bacteroides* contribute to the mental health of the host. Furthermore, since the intestinal mucus of the host is independent from nutrient uptake by the host, *Bacteroides* species can supply essential nutrients to the host during fasting time. These properties make *Bacteroides* species the next-generation probiotics [20]. 

The mechanism of mucin degradation has only recently been identified [21], when several O-glycanase from the family of glycoside hydrolase 16 (GH16) have been found to degrade the side chains of animal and human mucins. Identification of these enzymes is highly significant since it may help to identify potential differences between the O-glycans present in different intestinal diseases [21]. In addition, the ability to abrade mucin enables newborn colonization of the gut microbiota, with the associated advantage of an efficient microbiota when adult and the survival of the microbiota during the absence of glycans in the host diet [22]. The same study reports that excessive mucin degradation facilitates pathogen infection, inflammatory bowel disease (IBD) and colorectal cancer.

## 4. How Gut Microbiota Models the Immune System

**Figure 2:**
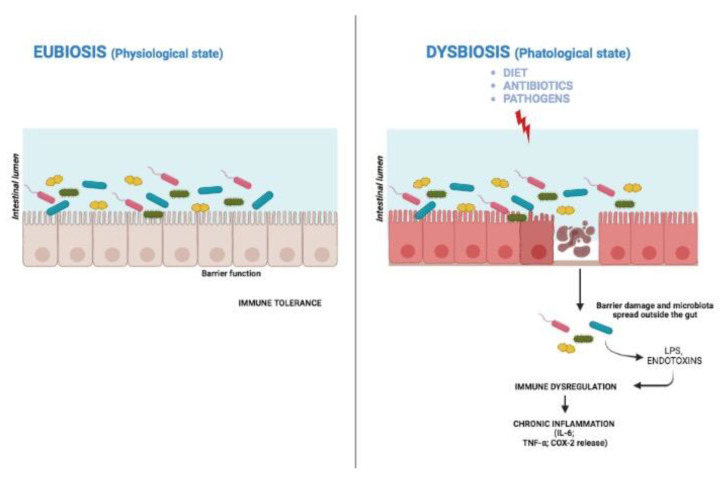
In eubiosis, the immune system tolerates the microbiota. Factors such as diet, antibiotics and microbial infections may cause a condition of dysbiosis that leads to diseases.

The tight coevolution between gut microbiota and the host requires that the immune system must recognize the gut microbiota as self. However, diet, early exposure to antibiotics, or pathogen infections might spread the microbiota outside the gut. In this case, the immune response—in the attempt to contain the diffusion of microbiota outside the gut—may favor inflammatory bowel disease (IBD), metabolic syndrome [23], neurodegenerative diseases [24], and cancer [25] (Figure 2). Thus, the interactions between gut microbiota and immune system are multiple, complex, and difficult to control.

The impact of the gut microbiota on the immune system is particularly determinant during the first year of life, when the microbiota composition is highly variable, and the immune system is still incomplete. This explains why newborns are highly susceptible to intestinal infections. In newborns, gut colonization starts soon after the delivery and reflects that of the mother. Maternal antibodies transmitted to the newborn baby via breast milk are essential to prevent pathogen infections [26]. Studies in GF mice have demonstrated that absence of gut microbiota inhibits the correct development of the lymphoid tissue. B cells play an important role in protecting the gut microbiota homeostasis producing a large quantity of IgA antibodies [26]. 

Babies—born by caesarean section or with a normal birth—during the first month of life live immaturely outside of the uterus and their digestive systems are also immature. During this time, the intestinal wall is exposed to bacteria, which are indispensable, but potentially dangerous at the same time. Babies born prematurely are at risk of necrotizing enterocolitis. However, provision of lactobacilli can prevent this risk by alerting the intestinal epithelium and the immune system against the presence of pathogens. Following birth, the microbiota of the baby is also under the influence of the environment. Breast milk provides optimal nutrition to the baby and its microbiota. Breast milk substitutes do not contain bioactive molecules or nutrients useful to the baby. This may influence the microbiota of the preterm-born baby, making it difficult to know whether the microbiota alteration of the preterm baby is attributable to the preterm birth or insufficient milk substitutes.

The intestine hosts two functionally distinct TH17 cell populations. The TH17 cells elicited by segmented filamentous bacteria (SFB) are not inflammatory; those elicited by *Citrobacter* are strongly inflammatory [27]. TH17 cells are absent in GF mice, but rapidly appear upon microbial colonization, and a bacterial polysaccharide isolated from *Bacteroides* fragilis corrects T cell deficiency in lymphoid tissues. Finally, gut microbiota diversity during infancy protects from mucosal IgE induction, which predisposes to allergy [28]. In conclusion, gut microbiota integrity protects against infection, allergy and inflammatory diseases during infancy and adulthood.

## 5. Gut Microbiota–Cystic Fibrosis Interaction

**Figure 3:**
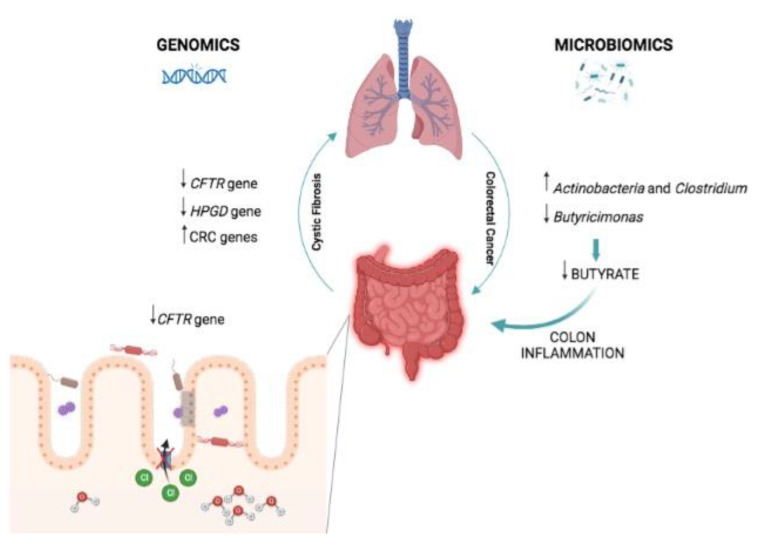
Prevalence in the microbiota of *Actinobacteria* and *Clostridia* and reduced level of butyrate-producing bacteria, along with downregulation of the host *CFTR* gene and *HPGD* genes predisposed to CF, which may lead to CRC.

Cystic fibrosis (CF) patients have a 5–10-fold higher risk of developing colorectal cancer (CRC). The first clue that CF and CRC might share several risk factors was finding that the genome of CF patients is enriched in CRC genes controlling immune response, cell adhesion, and viral infection. Further, the taxa of gut microbiota associated with CF (*Actinobacteria* and *Clostridium*) are enriched in CRC patients as well [29]. Furthermore, the two tumor suppressor genes, *CFTR* and *HPGD*—known to predispose to colorectal cancer—result in the downregulated colon of CF patients, who display an increased level of *Actinobacteria*, but a decreased level of butyrate-producing bacteria (such as *Butyricimonas*). Butyrate promotes growth and exerts anti-inflammatory activity, both highly important properties protecting the colon [30]. Reduced level of butyrate is also found in CRC patients [29]. In conclusion, though all the pathogenetic mechanisms of CF at present are not clear, the above studies show that the host gut microbiota—inducing high levels of pathogenic bacteria (*Ruminococcaceae*) and low levels of butyrate—has a decisive role in promoting carcinogenesis in the gut of CF patients (Figure 3). The data reported above might facilitate the discovery of new drugs against CRC and be used as potential biomarkers for an early detection of CRC in CF patients.

## 6. Gut Microbiota–Diet Interactions

**Figure 4:**
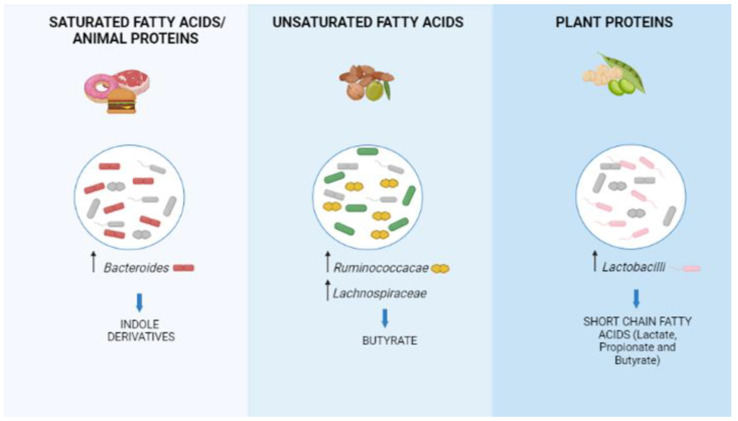
Influence of diet on microbiota composition. Saturated fatty acids and animal protein consumption increases the risk of cardiovascular diseases; unsaturated fatty acids and plant protein consumption protect against inflammation.

Twin studies have shown that host genes contribute to human gut microbiota diversity. However, environmental factors (principally, diet, and antibiotics) remain the principal determinants of diversity across individual human microbiota [31]. Diets rich in saturated fat promote expansion of *Bilophila wadsworthia*, which induces a pro-inflammatory response and colitis in *IL10 -/-*, but not in wild-type mice. Bile, an exclusive property of vertebrates, confers to the host the advantage of extracting more nutrients from the diet. However, bile is also a strong antimicrobial and thus can exclude from or include new components in the microbiota. At the same time, numerous intestinal pathogens are bile-resistant and highly favored in the presence of bile. In a predisposed host, even one single new pathogen species and its secondary bile acids can cause a chronic disease, in synergism with the other components of the microbiota. These results plausibly explain why Western diets rich in saturated fat may cause bowel disease in genetically predisposed hosts. In humans, consumption of diets high in saturated fatty acids are associated with high levels of *Bacteroides* and indole metabolites, both associated with cardiovascular diseases [32]. Diets rich in saturated fat lead also to immune dysregulation, which promotes chronic inflammation and development of numerous diseases, including type 2 diabetes; obesity; cardiovascular disease; hypertension; and cancer of the colon, breast, and prostate. The omega-3 polyunsaturated fatty acids instead increase the abundance of bacterial species producing butyrate. These results concur with the known anti-inflammatory and anti-cancer properties of omega-3 polyunsaturated fatty acids. In humans, a prolonged diet rich in animal proteins increases the abundance of *Bacteroides* [33], while a brief diet rich in animal proteins increases the abundance of bile-resistant bacterial species. In contrast, consumption of plant protein diets increases the abundance of lactobacilli and short-chain fat acids (Figure 4). The side effects of carbohydrates on the gut microbiota are complex and at present little is known about them.

## 7. Metabolites Derived from Gut Microbiota Have a Role in Diseases

Metabolites can pass through the gut barrier and reach the circulation [34]. In the liver, several TLRs recognize bacterial ligands inducing inflammation, which in turn contribute to induce non-alcoholic fatty liver disease (NAFLD) [35]. At the same time, NRRP6 and NLRP3 protect from NAFLD modulating the gut microbiota [36]. Interactions between the gut microbiota and the host immune system also contribute to diabetes. Non-obese and non-diabetic GF mice lacking MyD88 develop type 1 diabetes; the same mice—following microbial colonization—show an attenuated form of the disease. Depletion of *Akkermansia muciniphila* activates innate pancreatic β1a cells, inducing insulin resistance [37], while metabolites of tryptophan derived from the microbiota modulate white adipose tissue inflammation in obese patients via the miR-181 family of microRNA [38]. In mice, the innate immune sensor NLRP12 attenuates the effects of high fat diets, preserving the short chain fatty acids produced by bacteria of the *Lachospiraceae* family [39]. The gut microbiota-derived metabolite trimethylamine-N-oxide (TMAO) is associated with the atherosclerotic heart disease, in mice and in humans [40]. Interestingly, TMAO augments atherosclerosis upregulating the macrophage scavenger CD36 and SR-A1 and favoring the accumulation of cholesterol in macrophages. In conclusion, recent studies revealed the existence of a tight cooperation between gut microbiota and immune response. However, because of the high complexity of both systems, a clear causal link between them has not emerged yet. There is confidence that artificial intelligence may help deciphering the immune response of the host from his microbiota in the near future [10].

*Weissella confusa* DD_AT (*W. confusa*) displays anti-bacterial and immune-stimulatory activity in vivo [41]. *W. confusa* is present in the microbiota of zebrafish, mice, and humans [42]. There is a growing number of multidrug-resistant and pathogenic bacterial species solicited to test the anti-bacterial activity of *W. confusa* against *E. coli* 0157-H7. To test the potential antimicrobial activity of *W. confusa*, the zebrafish larvae were co-cultured with *E. coli* 0157-H7 and non-pathogenic *W. confusa*. *W. confusa* significantly reduced the number of *E. coli* in the pre-treated larvae. This study seems to us appropriate for mention. *W. confusa* encapsulated in nanoparticles might be used to control *E. coli* o157-H7 in the future.

## 8. Differently Expressed Genes in Gastric Cancer and Normal Tissue at Population Level

Worldwide, gastric cancer is the fourth cause of cancer and the annual number of cases of this form of cancer is about one million. The great majority of gastric cancer cases—but not all—are attributable to *Helicobacter pylori* (*Hp*). Gastric cancer proceeds through the steps of atrophic gastritis, intestinal metaplasia, and finally of gastric cancer. The current survival at 5 years of gastric cancer patients is less than 30%. This low survival rate is in part attributable to the heterogeneity of this type of cancer, which makes difficult to derive an early diagnosis. In particular, gastric microbiota and host gene expression vary during the disease process. Understanding these changes might help to better understand the pathogenesis and optimize immunotherapy, which is at present the standard treatment for gastric cancer. In this context, the identification of molecular biomarkers would be particularly valuable. Several methods attempted to identify differentially expressed genes (DEGs) in gastric cancer and normal tissues at the population level. Recently, an algorithm (named Rank Comp) solved the problem of using normal controls that recognize a very large collection of normal tissues. This algorithm has detected 25 universal DEGs specific to gastric cancer in the absence of paired normal tissue. The 25 DEGs—which include 12 upregulated and 13 downregulated genes—are present in 90% of the 1.090 gastric cancer biopsies and 90% of the 448 cancer-normal tissues; at present they are universal DEGs for gastric cancer. Furthermore, the 12 upregulated genes and the corresponding 284 linked genes (total of 296 genes) are enriched with 56 pathways associated with gastric cancer genes and are registered in COSMI C. The 13 downregulated genes and the 16 linked genes (total 29 genes) are enriched with 15 pathways associated with gastric cancer genes [43].

## 9. Lung Cancer: A Model to Study the Impact of Intestinal and Lung Microbiota in Carcinogenesis, Progression, and Response to Treatments

Local and systemic chronic inflammation cause cancer development and progression. Cytokines, chemokines and other pro-inflammatory factors can facilitate tumor growth and spread. The microbiota may also lead to the development and progression of cancer via (1) direct mutagenesis or regulation of oncogenic pathways and (2) modulation of host immune system [44]. Experiments in animal models provide significant insights to understanding these phenomena. Using conditional alleles of *KrasLSL-G12D* and *p53flox/flox*, KP mice develop spontaneous lung adenocarcinoma. Lung microbiota of KP mice includes *Staphylococcus, Streptococcus, Lactobacillus*, and *Pasteurellaceae* in both healthy and tumor-bearing mice, but when lung cancer develops, a significant increase emerges in the total bacterial burden and a reduced bacterial diversity in the airway. Furthermore, several bacterial species, including *Herbaspirillum* and *Sphingomonadaceae*, are more represented in tumor-bearing lungs, whereas others (*Aggregatibacter* and *Lactobacillus*) are more frequently found in lungs without tumor. If grown in germ-free conditions, or if polyantibiotherapy (ampicillin, vancomycin, neomycin, and metronidazole) is administered, strong protection against tumor development is observed [45]. Correlation between the microbiota and tumor development is local, as if antibiotic treatment is administered to only partially eliminate the commensal bacteria and bacterial burden in the lung; fecal bacterial load, in fact, is not correlated with tumor burden [45]. Yet, treating mice with metronidazole alone effectively suppresses lung tumor growth without reducing the overall bacterial abundance in the gut. Mechanistically, local microbiota stimulates MyD88-dependent IL-1β and IL-23 production from myeloid cells, inducing proliferation and activation of Vγ6 + Vδ1+ γδ T cells that produce IL-17 and other effector molecules to promote inflammation and tumor cell proliferation.

What about humans? Overall, the lung microbiota in the healthy human lung is composed predominantly of *Streptococcus Prevotella, Haeamophilus, Neisseria*, and *Fusobacterium* [46]. However, in lung diseases such as chronic obstructive pulmonary disease (COPD) or cancer, lung microbiota is dysregulated; in particular, in patients with lung cancer, microbiota seems enriched in terms of abundance, but reduced in terms of diversity [46]. Data from the PathSeq analysis of the Cancer Genome Atlas (TCGA) project in human tumors strongly support these experimental findings. This project identifies a number of bacterial taxa that are significantly enriched or depleted in squamous cell carcinoma and adenocarcinoma primary lung tumors as compared to normal lungs [45].

Apart from the above-quoted relationships between local microbiota and the immune system, other mechanisms may cause microbiota alteration, contributing to initial carcinogenesis and progression. Release of free radicals from the host immune response to bacteria may damage DNA and proteins. Thus, if cancer cells develop, their growth may be sustained by bacterial components, such as lipopolysaccharides (LPS), which have been shown to promote human epithelial non-small lung cancer cell (NSCLC) proliferation in vitro and in vivo by stimulating Cox-2 and prostaglandin pathways, an effect dependent on CD14 and Toll-like receptors (TLR-4). In the case of gram-positive bacteria, such as *Staphylococcus aureus*, lipoteichoic acid stimulates human NSCL cell growth interacting with TLR-2 that increases IL-8 secretion [47]. Furthermore, commensal microbiota has been shown to engage host cell Wnt/beta-catenin pathway, whose altered signaling can promote transcription of C-Myc and Cyclin D1oncogenes.

There is also evidence that microbiota may exert beneficial effects: epithelial lung cancer other than non-small cell lung cancer (NSCLC) patients treated with antibiotics prior to or during treatment with immune checkpoint inhibitors have a poorer clinical outcome [48]. Routy B. et al. also showed that antibiotic consumption is associated with poor response to programmed death-ligand 1 (PD-L1) immunotherapy [49]. They profiled intestinal microbiota and found that non-responding patients have low levels of *Akkermansia muciniphila* [49]. In the animal model, oral bacteria supplementation to antibiotic-treated mice restores the responses to immunotherapy [49]. 

Combined analysis of lung and gut microbiota in patients with NSCLC treated with immune checkpoint inhibitors have been evaluated recently with respect to clinical response [50]. *Fusobacteriaceae* in the gut and *Proteobacteria* in the lung are more abundant in non-responders, while *Bacilli* or *Streptococcus* are more abundant in responders [50]. All these findings strongly support the concept that both lung and intestinal microbiota play a significant role in lung cancer occurrence and progression as well as in response to treatments.

## 10. Role of Gut Microbiota in Gastric Cancer

Following the introduction of culture-independent approaches—including next-generation sequencing (NGS), fluorescent in situ hybridization (FISH), and dot-blot hybridization analysis, the dogma that the stomach is sterile because of its harsh acidity has been abandoned. At present, it is clear that the stomach holds a dense and diverse microbiota. An intense communication among different microbial populations and the host occurs through metabolically active molecules and gene regulation [51].

The main phyla in the stomach microbiota of *H. pylori*-negative subjects are *Actinobacteria, Bacteroidetes*, and *Firmicutes* [52]. *H. pylori* has been shown to be responsible for the alteration of the alpha-diversity of the normal microbiota of the stomach, leading to dysbiosis, which plays a key role in many diseases of the stomach, including primary cancer. *H. pylori* infection accounts for 40% to 90% of the gastric microbiota in infected individuals. Besides the downregulation of the microbial alpha-diversity found in individuals with chronic *H. pylori* gastritis and atrophic gastritis [53], the *H. pylori* cytotoxin-associated gene A (CagA), is responsible for microbial dysbiosis through changes in the gastric environment [54]. Additional mechanisms leading to the modification of the gut microbiota are elevation of gastric pH [55] and changes in the host diet [56].

All these radical changes induced in the gut microbial community involve not only gastric cancer but also several digestive diseases. *H. pylori* is involved in three quarters of gastric cancer cases including gastric adenocarcinoma, primary gastric lymphoma, and MALT lymphoma, which accounts for about 30–40% of extra nodal lymphomas [56]. The process of *H. pylori*-linked gastric carcinogenesis is a complex sequence of events that starts with the induction of a chronic inflammatory response in the gastric mucosa and culminates in the loss of acid-secreting parietal cells, which leads in turn to an increased gastric pH and dysbiosis. *H. pylori* colonization of the gastric mucosa requires several adherence factors such as BabA, SabA, OipA, and especially the virulence factor CagA: the strains expressing CagA are associated with high grades of gastric mucosa inflammation, chronic gastritis and gastric atrophy [57]. During *H. pylori* infection, the vacuolating cytotoxin A (VacA) favors the accumulation of CagA into the host cells and cag pathogenicity island (cagPAI) encoding the type IV secretion system (T4SS) that facilitates the entry of CagA in the gastric mucosa cells [55,57,58]. *H. pylori* also releases lipopolysaccharide (LPS) and surface proteins within the lamina propria of the stomach with consequent increased macrophage secretion of interleukin 1β (IL-1 β), IL-8, IL-17, and tumor necrosis factors-α (TNF- α), which are implicated in the process of gastric carcinogenesis. The aggressive stimulation of the innate and adaptive host immune response that follows *H. pylori* colonization of the stomach may result in elimination of the pathogen or its chronic infection that leads to the well-known sequence of chronic gastritis, which in the long-term may evolve into gastric atrophy, intestinal metaplasia, dysplasia, and intestinal-type gastric carcinoma. However, hypochlorhydria associated with chronic *H. pylori* infection—due to destruction of parietal cells in the gastric mucosa—leads to the progressive decline of *H. pylori* infection and the gastric colonization by other digestive tract bacteria leading to dysbiosis. The latter include multiple non-*H. pylori* species such as *Streptococcus* spp., *Lactobacillus* spp., *Xanthomonas* spp., *Proteus* spp., *Klebsiella* spp., *Pseudomonas* spp., *Neisseria* spp., *E. coli*, and *Campylobacter jejuni* [59]. Gastric carcinogenesis may then be promoted by non-*H. pylori* bacteria through the production of metabolites, including N-nitroso compounds and lactate but also through the induction of chronic inflammatory response, modulation of immune response, and DNA damage. 

While there is evidence that the gastric cancer risk increases as result of the cooperation between *H. pylori* and non-*H. pylori* bacteria, the precise mechanisms responsible for this increased risk are still not clear [60].

Gut microbiota plays a major role on the maturation, development and function of multiple populations of host gut immune cells. This is why germ-free mice show deficient development of gut-associated lymphoid tissues (GALT), Peyer’s patches, mesenteric nodes, and decreased immune cells in the small bowel cellular lamina propria. Epithelial cytokine expression controlling the activity of macrophages, T and B lymphocytes, are influenced by the microbiota [61]. Also, the inflammatory or homeostatic state in the gut mucosa is the net result of the expression of pro-inflammatory (IL-1b, TNF-a, IL-2, IL-6, IL-15, IL-21, IL-23) and anti-inflammatory cytokines (IL-10 and TGF-b) [62]. The process of malignant transformation and progression and the modulation of the immune response are intimately linked. The alteration of gut microbiota may influence the host immune response activating inflammatory-dependent mechanisms that trigger cell proliferation and decrease apoptosis.

Short chain fatty acids (SCFAs)—including butyrate, acetate, and propionate—secreted by the gut microbiota may prevent malignant transformation through several mechanisms. SCFAs can act directly on cancer cells by blocking histone deacetylases (HDACs), which leads to reduced expression of pro-inflammatory cytokines (IL-6 and NF-kB). Furthermore, they prevent the production of dendritic cells mediating inflammatory reactions and promote the development of Tregs controlling inflammation and malignant transformation. Metabolites produced by commensal bacteria promote peripheral regulatory T cell generation [63], downregulation of pro-inflammatory cytokines, and upregulation of IL-10 and TGF-β, both with immunosuppressive properties. SCFAs can also interact with the immune system directly by interacting with G-protein-coupled receptors (GPCRs), which results in increased expression of IL-10 and transforming growth factor-beta (TGF- β), decreased expression of macrophages and neutrophils pro-inflammatory cytokines, and blockade of T helper type 17 (Th17) cell differentiation [64]. SCFAs also participate in the activation of the inflammasome and peroxisome proliferator-activated receptor-*γ* (PPAR-*γ*) pathway, which results in increased mucin secretion favoring epithelial integrity [65].

Natural killers (NKs) are lymphocytes of the innate immune system devoted to preventing the development and progression of cancer cells and are the target of immune checkpoint inhibitor drugs and monoclonal antibodies [66]. The accumulation of NK cells promoted by a favorable microbiome is an interesting and promising therapeutical target, as fecal microbiota transplantation is now actively used in clinical current practice although with different indications [67].

In recent years the introduction of checkpoint inhibitor immunotherapy drugs that block proteins suppressing T cell responses as Cytotoxic T-lymphocyte-associated protein 4 (CTLA-4), programmed death-ligand 1 (PD-1) and its ligand PD-L1 have radically changed the treatment approach and prognosis of many cancers. Recently, it has been shown in the rodent that gut microbiota can enhance the effectiveness of cancer immunotherapy [68]. These findings open new research horizons on the role of microbiota in cancer immunotherapy.

## 11. Potential Role of Anti-Inflammatory Cytokines against Cancer

Infection, innate immunity, chronic inflammation, and cancer have cytokines and molecules produced by cancer cells as a common link, which activate inflammatory cells and tumor growth. Independent studies have also found a tight interaction between chronic infection and cancer [69], as between *Epstein Barr* virus (EBV) infection and Burkitt lymphoma [70]. It has also been found that bacterial infections caused by surgical complications promote rapid development of metastasis in mice [71] and in humans [72]. Indeed, infections promote inflammation through the activation of receptors recognizing pathogens (pathogen-associated molecular patterns; PAMPS), as bacterial cell wall components or DNA. Generally, pro-inflammatory cytokines produced by host-immune cells or tumor cells promote tumor development. However, the anti-inflammatory cytokines Il-10 and TGF-β inhibit tumor development [70]. This result suggests a new therapeutic approach against the cancer, based on the inhibition of pro-inflammatory and tumor-promoting cytokines and the enhancement of the activity of the anti-inflammatory cytokines [70,73].

## 12. Conclusions

The present study shows how the intestinal microbiota exerts a profound effect on numerous metabolic diseases. Here we mention only two diseases, both common: colorectal cancer (CRC), and hepatocellular carcinoma (HCC). CRC and HCC are the second and third causes of death by cancer, respectively. What we say about the primary cause of these two metabolic diseases holds for the others as well. Primary causes of CRC and HCC are abnormal use of alcohol, smoking, type 2 diabetes, and obesity. Altered gut microbiota—leading to intestinal inflammation and dysbiosis—is the factor common to both diseases. Dysbiosis may emerge from the loss of beneficial bacteria, abundance of pathobionts (opportunistic bacteria coming out as result of perturbation of the health microbiota), or reduced diversity of the microbiota. From the above facts it is clear that prevention of intestinal microbiota alteration is the best approach to the control of the above diseases (and the many more that the collective effort of multiple competencies will discover in the near future). In the meantime, there is a need to translate what is already known into clinical medicine, essential methods that consent the rapid analysis of the complex data emerging from the study of intestinal microbiota to identify the components contributing to the modelling of the microenvironment of each disease [74].

## Data Availability

Not applicable.
